# Chemical Profiling Provides Insights into the Metabolic Machinery of Hydrocarbon-Degrading Deep-Sea Microbes

**DOI:** 10.1128/mSystems.00824-20

**Published:** 2020-11-10

**Authors:** Aldo Moreno-Ulloa, Victoria Sicairos Diaz, Javier A. Tejeda-Mora, Marla I. Macias Contreras, Fernando Díaz Castillo, Abraham Guerrero, Ricardo Gonzalez Sanchez, Omar Mendoza-Porras, Rafael Vazquez Duhalt, Alexei Licea-Navarro

**Affiliations:** a Departamento de Innovación Biomédica, Centro de Investigación Científica y de Educación Superior de Ensenada, Baja California (CICESE), Ensenada, México; b Consorcio de Investigación del Golfo de México (CIGOM), CICESE, Ensenada, México; c Departamento de Bionanotecnología, Centro de Nanociencias y Nanotecnología, Universidad Autónoma de México, Ensenada, México; d Cátedras CONACyT-CIAD/Centro de Investigación en Alimentos y Desarrollo, A.C. Unidad Mazatlán, Sinaloa, Mexico; e CSIRO Livestock and Aquaculture, Queensland Bioscience Precinct, St Lucia, QLD, Australia; State University of Maringá

**Keywords:** 16S rRNA, deep-sea microbes, hydrocarbon degradation, marine bacteria, mass spectrometry, metabolomics

## Abstract

Marine microbes are known to degrade hydrocarbons; however, microbes inhabiting deep-sea sediments remain largely unexplored. Previous studies into the classical pathways of marine microbial metabolism reveal diverse chemistries; however, metabolic profiling of marine microbes cultured with hydrocarbons is limited. In this study, taxonomic (amplicon sequencing) profiles of two environmental deep-sea sediments (>1,200 m deep) were obtained, along with taxonomic and metabolomic (mass spectrometry-based metabolomics) profiles of microbes harbored in deep-sea sediments cultured with hydrocarbons as the sole energy source. Samples were collected from the Gulf of México (GM) and cultured for 28 days using simple (toluene, benzene, hexadecane, and naphthalene) and complex (petroleum API 40) hydrocarbon mixtures as the sole energy sources. The sediment samples harbored diverse microbial communities predominantly classified into *Woeseiaceae* and *Kiloniellaceae* families, whereas *Pseudomonadaceae* and *Enterobacteriaceae* families prevailed after sediments were cultured with hydrocarbons. Chemical profiling of microbial metabolomes revealed diverse chemical groups belonging primarily to the lipids and lipid-like molecules superclass, as well as the organoheterocyclic compound superclass (ClassyFire annotation). Metabolomic data and prediction of functional profiles indicated an increase in aromatic and alkane degradation in samples cultured with hydrocarbons. Previously unreported metabolites, identified as intermediates in the degradation of hydrocarbons, were annotated as hydroxylated polyunsaturated fatty acids and carboxylated benzene derivatives. In summary, this study used mass spectrometry-based metabolomics coupled to chemoinformatics to demonstrate how microbes from deep-sea sediments could be cultured in the presence of hydrocarbons. This study also highlights how this experimental approach can be used to increase the understanding of hydrocarbon degradation by deep-sea sediment microbes.

**IMPORTANCE** High-throughput technologies and emerging informatics tools have significantly advanced knowledge of hydrocarbon metabolism by marine microbes. However, research into microbes inhabiting deep-sea sediments (>1,000 m) is limited compared to those found in shallow waters. In this study, a nontargeted and nonclassical approach was used to examine the diversity of bacterial taxa and the metabolic profiles of hydrocarbon-degrading deep-sea microbes. In conclusion, this study used metabolomics and chemoinformatics to demonstrate that microbes from deep-sea sediment origin thrive in the presence of toxic and difficult-to-metabolize hydrocarbons. Notably, this study provides evidence of previously unreported metabolites and the global chemical repertoire associated with the metabolism of hydrocarbons by deep-sea microbes.

## Abstract (precis)

High-throughput technologies and emerging informatics tools have significantly advanced knowledge of hydrocarbon metabolism by marine microbes. However, research into microbes inhabiting deep-sea sediments (>1,000 m) is limited compared to those found in shallow waters. In this study, a nontargeted and nonclassical approach was used to examine the diversity of bacterial taxa and the metabolic profiles of hydrocarbon-degrading deep-sea microbes. In conclusion, this study used metabolomics and chemoinformatics to demonstrate that microbes from deep-sea sediment origin thrive in the presence of toxic and difficult-to-metabolize hydrocarbons. Notably, this study provides evidence of previously unreported metabolites and the global chemical repertoire associated with the metabolism of hydrocarbons by deep-sea microbes.

## INTRODUCTION

The Gulf of Mexico (GM) is an environment occupied by numerous oil platforms and impacted by multiple accidental oil spills, including the Deepwater Horizon (DWH) (1) and Ixtoc-I (2) spills. Estimations suggest that deep-sea sediments down to a depth of 1,700 m were contaminated with oil after the DWH spillage (3). Deep-sea sediments are considered the largest ecosystems on Earth covering approximately 65% of the Earth’s surface (4) and harboring diverse and abundant microbial communities (5) capable of degrading hydrocarbons (6–8).

Microbial metabolism plays a pivotal role in the removal of oil from the ocean (6) as evidenced by a surge in oil-degrading bacteria (ODB) in deep-sea sediments following the DWH incident (9). Samples of surficial (0 to 3 cm) deep-sea sediments (>1,400 m depth), collected adjacent to the DWH wellhead 4 to 6 months after the DWH spill, were analyzed revealing a predominance of phylum *Proteobacteria* (7, 8). Also, an enrichment of genes associated with hydrocarbon degradation (i.e., aerobic and anaerobic pathways) was observed compared to samples collected away from the wellhead (7).

Previous studies reveal differences in bacterial classes in deep-sea sediments collected from various seafloor depths (0 to 1 cm versus 1.5 to 3 cm) (7, 8). Furthermore, a comprehensive study of more than 700 samples by Overholt et al. (5) reported the prevalence of phylum *Proteobacteria* in deep-sea sediments collected from 29 sites adjacent to the DWH (up to 2,290 m depth) and Ixtoc-I (up to 1,440 m depth) oil spills (5). A major determinant in the microbial community composition observed was the sediment depth (range, 0 to 15 cm below seafloor). Unfortunately, microbial metabolism was not reported in this study.

Hydrocarbon degrading potential of microbes from deep-sea sediments has been inferred mostly through metagenomics and amplicon sequencing analysis (e.g., 16S rRNA) (7, 10). DNA and RNA sequencing provide evidence of the genetic potential of microbial communities to produce molecules or metabolites (11), however, liquid chromatography mass spectrometry (LC-MS) allows direct metabolomic profiling and, in some cases, metabolite quantification (12). In combination, amplicon sequencing and LC-MS-based metabolomics could help to elucidate the functional state of microbes or communities under specific conditions.

LC-MS-based metabolomics is a powerful tool to profile the chemistry of microbial cultures (13). This technique generates large data sets containing hundreds to thousands of molecules that can be rapidly dereplicated (i.e., identification of previously characterized molecules) (14) when coupled to appropriate computational tools (15–17). This type of approach has been successfully implemented to globally visualize metabolomes of individual and grouped bacterial strains (13), study small molecules within a specific pathway (18), and perform targeted isolation of new chemical entities (19).

**FIG 1:**
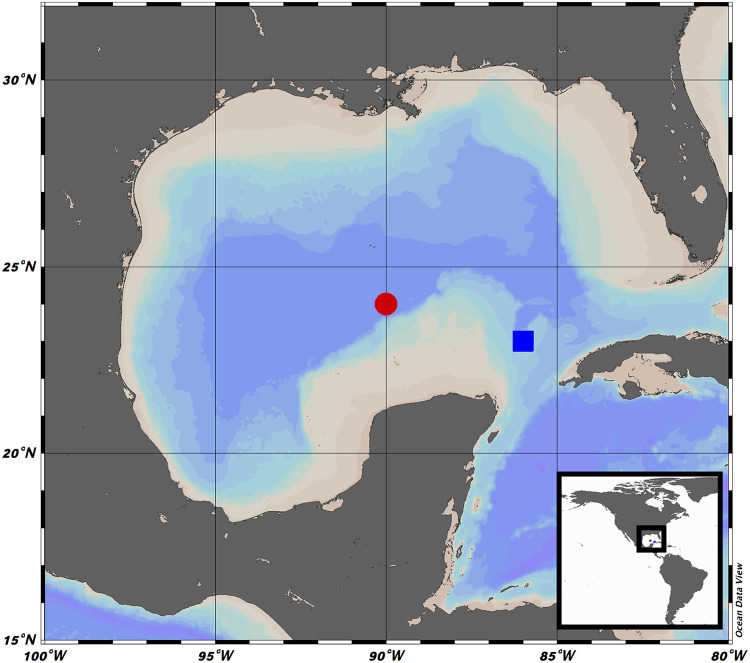
Sampling locations in the Gulf of México. Two superficial sediment samples (0 to 10 cm) were collected from water depths of 1,265 m (blue square, designated B18 [23°54′57.6″N, 86°47′28.8″W]) and 3,500 m (red circle, designated A7 [24°57′37.74″N, 90°0′52.5″W]). Maps were created with Ocean Data View software (version 5.1.5, http://odv.awi.de/).

**FIG 2:**
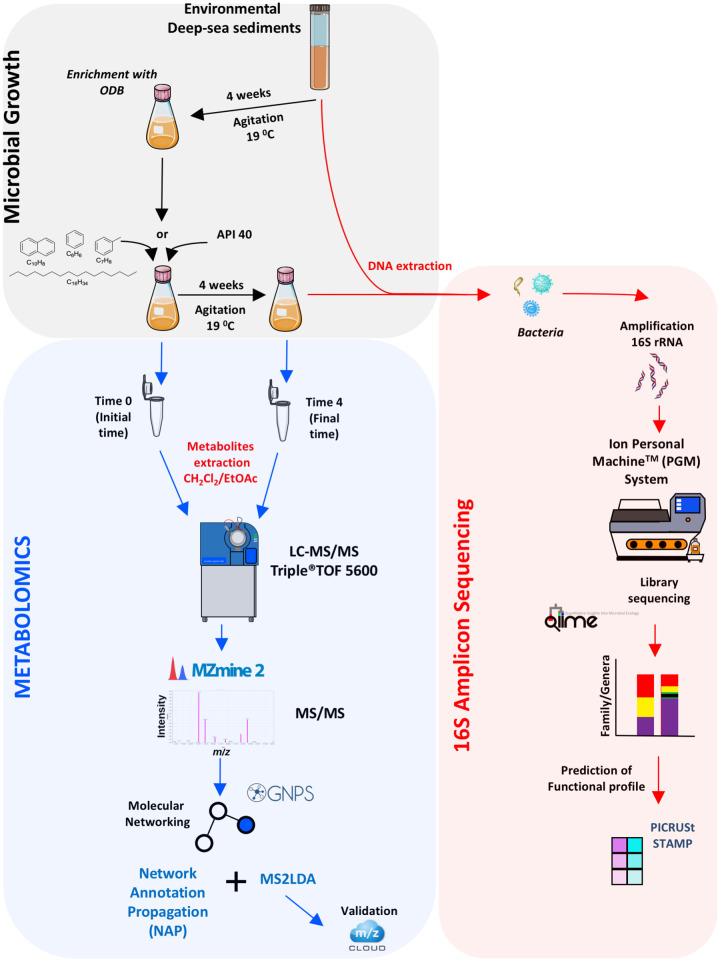
Illustration of study methodology. ODB, oil-degrading-bacteria; API 40, crude oil American Petroleum Institute 40 gravity.

In this study, the microbial community taxonomic composition and metabolome of two deep-sea sediments (>1,200 m deep), cultured with hydrocarbons as the sole energy source, were profiled (Fig. 1 and 2). To gain insight into the hydrocarbon metabolism of deep-sea microbes, we used a nonclassical and nontargeted approach comprised of molecular networking and complementary “state of the art” *in silico* dereplication strategies.

## RESULTS

### Growth of microorganisms from deep-sea sediments with hydrocarbons as sole energy source.

**FIG 3:**
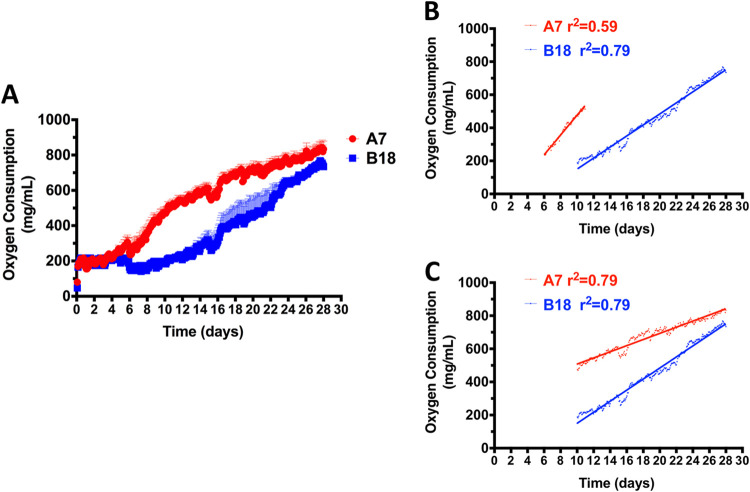
Hydrocarbon-enriched growth standardization of microorganisms from deep-sea sediments. (A) Microbial growth of A7 and B18 was evaluated in media containing 4 g liter^−1^ of sodium nitrate and 0.4% (vol/vol) API 40. Linear oxygen consumption within the growth curve was fitted with linear regression after a lag time of 0 to 6 days. (B) Linear regression analysis of the first exponential growth phase (6 to 10 days) of A7 compared to that of B18 (10 to 28 days). (C) Linear regression analysis of the second exponential phase of A7 (10 to 28 days) compared to that of B18 (10 to 28 days). Detailed calculations are provided in Results. All experiments were performed in triplicate.

Twelve deep-sea sediments were cultured with API 40 and screened for changes in oxygen consumption (see Fig. S1 in the supplemental material). Compared to other cultures, deep-sea sediments A7 and B18 showed an increase in oxygen consumption and were selected for further analysis. Growth of A7 and B18 with API 40, as per oxygen consumption rate, is shown in Fig. 3A. Significant differences in oxygen consumption rate between A7 and B18 were observed showing lag times of approximately 4 and 6 days. Findings also show that A7 appears to follow a two-step oxygen consumption pattern with two exponential phases, ranging from approximately 6 to 10 days (63.72 ± 4.28 mg/ml day^−1^) and 10 to 28 days (18.53 ± 0.36 mg/ml day^−1^), that were fitted with a linear regression (*r*^2^ > 0.59 and *r*^2^ > 0.79). Conversely, B18 shows a lag time of approximately 6 days and a unique exponential phase from 10 to 28 days (33.38 ± 0.65 mg/ml day^−1^) that were also fitted with a linear regression (*r*^2^ > 0.79). The first slope of A7 indicates a significantly higher oxygen consumption rate than B18 (63.72 ± 4.28 mg/ml day^−1^ versus 33.38 ± 0.65 mg/ml day^−1^, *P* < 0.0001) (Fig. 3B); however, after 10 days, A7 showed a lower growth rate compared to B18 (18.53 ± 0.36 mg/ml day^−1^ versus 33.38 ± 0.65 mg/ml day^−1^, *P* < 0.0001) (Fig. 3C). No significant oxygen consumption was observed in control groups (data not shown).

10.1128/mSystems.00824-20.2 FIG S1 Oxygen consumption of 12 deep-sea sediments cultured with API 40. Deep-sea sediments collected from the Gulf of Mexico were cultured with API 40 as the sole source of carbon for 28 days. Abiotic control: OxiTop flasks cultured without microbes but with all reagents. Deep-sea sediments are named arbitrarily. Graph lines represent the mean of triplicates for each condition. Oxygen consumption occurred only in cultures with deep-sea sediments A7 and B18, therefore these sediments were selected for further analysis. Download FIG S1, TIF file, 0.8 MB. Copyright © 2020 Moreno-Ulloa et al. 2020 Moreno-Ulloa et al. This content is distributed under the terms of the Creative Commons Attribution 4.0 International license.

### Microbial communities of environmental deep-sea sediments and API 40-enriched sediment samples.

Bioinformatics enabled the detection of 1,066 operational taxonomic units (OTUs) associated with bacteria and the classification of 132 up to genera. Most of the taxon detected in all combined samples were associated with the *Proteobacteria* (63.3%), *Firmicutes* (10.7%), *Acidobacteria* (9.5%), *Actinobacteria* (7.2%), *Gemmatimonadetes* (2.15%), *Nitrospirae* (2.09%), and *Bacteroidetes* (0.87%) phyla and others (see Table S1 in the supplemental material).

10.1128/mSystems.00824-20.7 TABLE S1 General list of phyla detected in all samples. Download Table S1, XLS file, 0.06 MB. Copyright © 2020 Moreno-Ulloa et al. 2020 Moreno-Ulloa et al. This content is distributed under the terms of the Creative Commons Attribution 4.0 International license.

**FIG 4:**
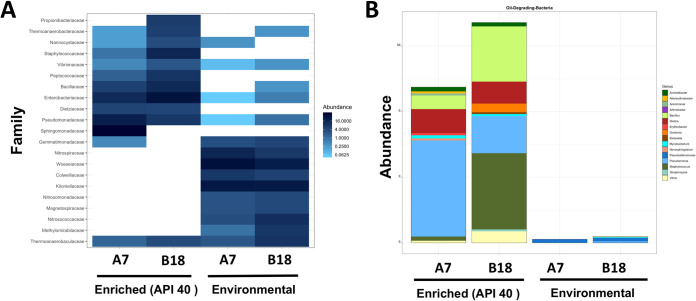
Bacterial community diversity in environmental and petroleum-enriched deep-sea sediments. (A) Microbial diversity at the family level. Only the 10 most abundant are defined. (B) Abundances of genera associated with oil-degrading bacteria are referred to as ODB.

In environmental deep-sea samples, 104 families were detected, with the most abundant families (in both A7 and B18) being *Woeseiaceae* (10.66%), *Kiloniellaceae* (8.82%), *Nitrospiraceae* (4.06%), *Colwelliaceae* (2.26%), *Nitrosococcaceae* (2.04%), *Thermoanaerobaculaceae* (1.61%), *Gemmatimonadaceae* (1.22%), *Methylomirabilaceae* (1.19%), *Nitrosomonadaceae* (1.06%), *Magnetospiraceae* (1.06%). The abundance of each remaining family was <1% (Fig. 4A). Consortium A7 showed higher diversity than B18 wherein 47 (45.19%) bacterial families were exclusively found in A7, compared to 9 (8.65%) unique to B18 samples. Forty-eight (46.15%) families were shared among groups. The alpha-diversity values measured by the Shannon index were 6.16 and 5.22 for A7 and B18.

In API 40-enriched samples, 44 families were detected with the most abundant bacterial families in both A7 and B18 being *Sphingomonadaceae* (20.93%), *Enterobacteriaceae* (16.84%), *Pseudomonadaceae* (10.15%), *Bacillaceae* (6.93%), *Staphylococcaceae* (6.13%), *Peptococcaceae* (3.84%), *Dietziaceae* (3.50%), *Propionibacteriaceae* (2.32%), *Thermoanaerobacteraceae* (2.11%), *Thermoanaerobaculaceae* (1.93%), *Nannocystaceae* (1.38%), and *Vibrionaceae* (1.03%). The abundance of each remaining family was <1% (Fig. 4A). Overall, 42 bacterial families (95.5%) were common to A7 and B18, while 2 (4.5%) were exclusive to A7. No exclusive families were detected in B18. The alpha-diversity index values were 3.3 and 3.1 for A7 and B18 and were lower than for their environmental counterparts.

Differences in the abundance of ODB were evaluated to gain further insight into microbial communities in deep-marine sediments (20). The combined output of all samples (deep-sea sediments and API 40 enriched) revealed 16 genera (*Pseudomonas*, *Staphylococcus*, *Bacillus*, *Dietzia*, *Vibrio*, *Gordonia*, Acinetobacter, *Pseudoalteromonas*, *Mycobacterium*, *Novosphingobium*, *Arenimonas*, *Erythrobacter*, *Altererythrobacter*, *Klebsiella*, *Streptomyces*, and *Arthrobacter*) linked to ODB. However, the presence and abundance of ODB varied according to the groups compared (Fig. 4B; see also Table S2). Five genera were detected in environmental samples (both A7 and B18), including *Pseudomonas*, *Bacillus*, *Pseudoalteromonas*, *Mycobacterium*, and *Arthrobacter*, and comprised 1.4% of total microbial abundance. Only *Pseudoalteromonas* and *Pseudomonas* were common among A7 and B18 groups, with *Pseudoalteromonas* displaying the highest abundance in both groups.

10.1128/mSystems.00824-20.8 TABLE S2 General list of genera detected in all samples. Download Table S2, XLS file, 0.07 MB. Copyright © 2020 Moreno-Ulloa et al. 2020 Moreno-Ulloa et al. This content is distributed under the terms of the Creative Commons Attribution 4.0 International license.

Genera identified in deep-sea sediments were also part of the ODB panel detected in A7 and B18 API 40-enriched groups, wherein the same five genera comprised 57.3% of the total microbial abundance (both A7 and B18). However, differences were noted when comparing A7 and B18 groups (Fig. 4B; see also Table S2). Overall, of the 16 detected genera, 11 (from 0.31 to 14.64% of relative abundance) were detected in A7, and 10 (from 0.29 to 11.62% of relative abundance) were detected in B18. *Pseudomonas*, *Bacillus*, and *Staphylococcus* were the dominant genera in B18, whereas *Pseudomonas*, *Dietzia*, and *Bacillus* were dominant in A7 (Fig. 4B).

### Prediction of metabolic function by PICRUSt.

**FIG 5:**
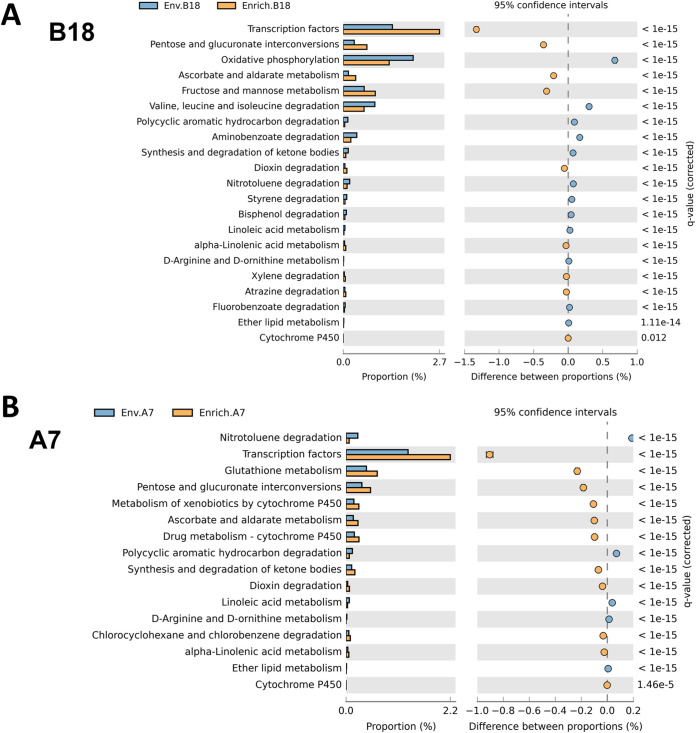
PICRUSt predictions of the functional profile of environmental and petroleum-enriched deep-sea sediments. (A) Comparison of B18 environmental and API 40-enriched samples. (B) Comparison between A7 environmental and API 40-enriched samples. Bars represent predicted KOs (KEGG, level 3) and associated proportion among samples. Only the most significant KOs are shown. Data from PICRUSt were imported into STAMP software for statistical analysis and visualization. Differences were assessed with the “two samples” analysis function included in STAMP (see Materials and Methods). Differences between proportions were considered significant if *P* value* *<* *0.05 using Fisher exact test.

To understand the metabolic potential of microbes from environmental and petroleum-enriched samples, functional profiles of 16S rRNA data were inferred using PICRUSt (21). In general, level 1 metabolism (amino acid and carbohydrate metabolism) was the most abundant KEGG orthology (KO) among the categorized functions, followed by level 2 metabolism (energy metabolism) (Fig. 5). Shifts in the functional categories linked to hydrocarbon metabolism and related metabolic pathways were then investigated in environmental and oil-enriched samples. Overall, a similar pattern was observed between the predicted functional categories of A7 and B18 enriched samples compared to counterparts. Following culture with hydrocarbons, A7 and B18 showed an increased representation of KOs (level 3, KEGG orthology) associated with alkane and aromatic degradation, including xylene, dioxin, atrazine, chlorocyclohexane, and chlorobenzene degradation, and cytochrome P450 (Fig. 5). However, other KOs related to hydrocarbon degradation (e.g., fluorobenzoate, nitrotoluene, and polycyclic aromatic hydrocarbon degradation) were more highly represented in environmental samples compared to enriched samples.

### Metabolites associated with hydrocarbon-degrading microorganisms from deep-sea sediments.

**FIG 6:**
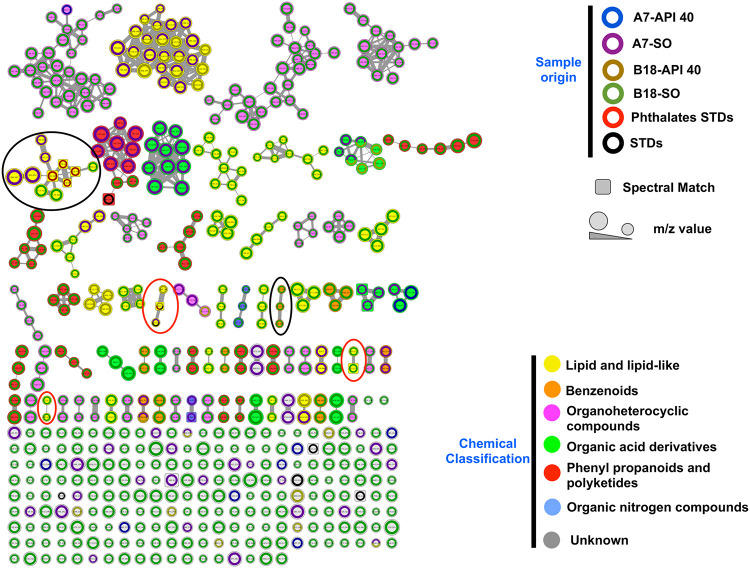
Molecular network of detected chemistries of deep-sea sediments grown with petroleum API 40 and synthetic oil (SO). Global mass spectral molecular network of A7 and B18 grown with SO and API 40 for 28 days. Each node represents a precursor ion (MS1), and edge thickness between nodes indicates similarity in MS2 fragmentation patterns. The node size and border color indicate the *m/z* value and type of hydrocarbon+microbial sample origin (SO or API 40), respectively. In addition, the geometrical figure (square) of the node indicates the use of an analytical standard or a spectral match (within GNPS spectral libraries). A total of 551 mass spectral nodes (over a mass range of 147.044 to 882.687 *m/z*) organized into 76 independent molecular families (with at least two connected nodes) were organized. The color of the node denotes the structural annotation of the global molecular network of A7 and B18 at the superclass level using NAP (ClassyFire classification). For each network with ≥2 nodes, a consensus candidate structure per node was assigned by NAP, and each structure was subsequently classified using ClassyFire, wherein the most frequent consensus classifications per network or cluster were retrieved to assign a putative superclass annotation to each network or cluster. Clusters with gray nodes indicate an unassigned superclass. Red ovals denote the clusters characterized with evidence of alkane hydrocarbons degradation. Black ovals denote the clusters characterized with evidence of aromatic hydrocarbons degradation.

The generated combined molecular network, or spectral network, comprised 551 mass spectral nodes (over a mass range of 147.044 to 882.687 *m/z*; Fig. 6) organized into 76 independent molecular families (with at least two connected nodes) (Fig. 6). The ion co-occurrence in two or more conditions is illustrated by a combination of colors in the node border. A total of 2.7% (*n* = 15) precursor ions cooccurred in two or more culture conditions, showing high variability in the production of secondary metabolites or hydrocarbon-derived metabolites between A7 and B18 microbes. The number of unique precursor ions was considerably higher for B18 grown with synthetic oil (SO), followed by A7 also grown with SO.

Spectral data were screened against public databases within the Global Natural Products Social Molecular Networking web platform (GNPS), leading to an automatic annotation (e.g., spectral match) of 1.8% (*n* = 10) of the total features analyzed (22). The data showed the diversity of metabolites predominantly belonging to the superclass of lipids and lipid-like molecules (21 networks) and next to the organoheterocyclic compound superclass (20 networks) (Fig. 6). The top five substructures annotated were related to metabolites containing a phthalic acid core, a nitrogen ring group, an underivatized carboxylic acid group, a hydroxyl group, and a primary amine with a hydroxyl group (Table 1). However, due to the challenging task of characterizing the entire metabolome of microbes derived from untargeted analysis, as well as the metabolic complexity of potential and not previously described microbes, NAP/MS2LDA-driven metabolite annotation was applied only to selected molecular networks (e.g., enriched-networks with standards or spectral library hits) as described by Kang et al. (23).

**TABLE 1:** Annotated Mass 2 Motif (M2M) findings with at least four metabolites by a match against public data sets within the GNPS platform and manual inspection using mzCloud (Search Peak tool) and METLIN databases (Neutral Loss Search tool)

M2M	Annotation	No. of metabolites	Precursor masses [M+H]^+^, *m/z*
M2M_260	Phthalate related	15	882.687, 854.654, 574.336, 557.312, 502.228, 464.375, 453.284, 391.285, 360.125, 313.144, 280.172, 279.16, 224.069, 223.096, 205.122
M2M_18	C_2_H_3_N loss	10	408.184, 380.153, 380.121, 378.13, 376.122, 348.09, 334.075, 327.14, 284.058, 267.081
M2M_38	Loss of CH_2_O_2_ indicative for underivatized carboxylic acid group	10	349.164, 309.134, 300.16, 259.167, 250.087, 249.112, 224.069, 213.091, 177.092, 153.091
M2M_43	H_2_O loss	10	394.203, 363.208, 351.181, 349.164, 333.17, 323.114, 316.322, 288.29, 187.143, 177.092
M2M_360	Loss of NH_3_ adducts + H_2_O	10	686.394, 544.331, 514.322, 502.228, 470.295, 456.28, 422.237, 382.245, 368.207, 276.254
M2M_1	Sterone related	9	605.305, 585.302, 536.243, 465.222, 380.121, 376.122, 358.202, 348.09, 345.228
M2M_12	Leucine related fragments and losses	9	526.222, 427.142, 414.077, 394.108, 378.207, 309.158, 265.142, 251.128, 237.112
M2M_212	Multiple cores-CH_4_SO loss	9	300.196, 288.199, 274.182, 260.168, 246.151, 232.136, 190.123, 187.115, 185.098
M2M_451	Linear dicarboxylic acid-related structure	7	539.426, 500.238, 466.371, 294.06, 206.138, 203.128, 189.113
M2M_56	Double H_2_O loss	7	369.161, 251.2, 244.191, 203.106, 191.106, 185.153, 179.107
M2M_73	Oxyacetyl-amino-methyl-cyclohexane-1-carboxylic acid-related structure	6	585.302, 583.309, 545.298, 536.243, 459.205, 358.202
M2M_295	Small peptidic substructure	5	373.281, 359.183, 333.275, 232.201, 187.143
M2M_39	Steroid backbone	4	269.2480, 239.2, 191.1430, 185.1530
M2M_37	Fragments indicative for cinnamic/hydroxycinnamic acid substructure	4	550.1280, 462.19, 462.076, 414.077
M2M_49	Loss possibly indicative of carboxylic acid group with one carbon attached	4	249.112, 185.153, 183.138, 179.1070
M2M_115	CHOOH loss-indicative for free carboxylic acid group	4	366.207, 334.1440, 293.211, 283.117
M2M_17	C_4_H_8_ loss indicative for saturated C_4_-alkyl substructure	4	352.169, 335.212, 333.222, 300.196
M2M_119	Loss of hexanoic acid	4	345.228, 311.15, 237.17, 185.1530

**FIG 7:**
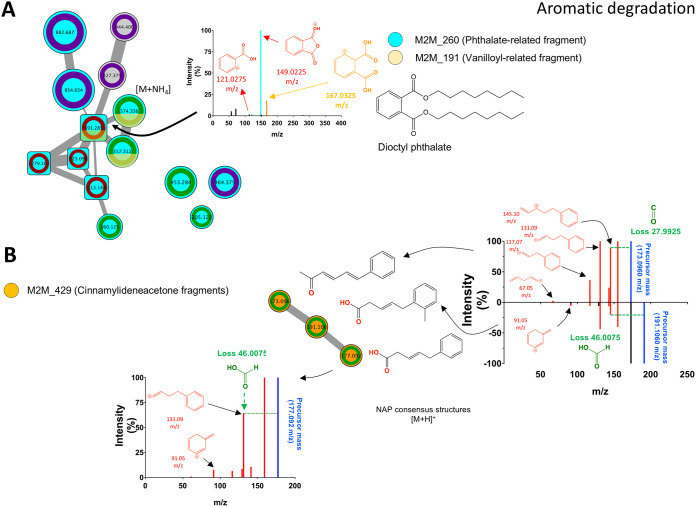
NAP/MS2LDA/mzCloud-driven metabolite annotation of selected chemistries linked to aromatic degradation. (A) Phthalic acid-related M2M annotated by analytical phthalates standards (used to enrich the global network). Fragments 121.0275, 149.022, and 167.0325 *m/z* (M2M_260 and M2M_191) were assigned to a phthalic acid-core (yellow substructure) using Heuristic fragmentation prediction by mzCloud (www.mzcloud.org). (B) Cluster containing the M2M_429 linked to cinnamylideneacetic acid. Chemical structures drawn here are the top-ranked consensus candidates predicted by NAP. Neutral losses are depicted in green, and fragments are depicted in red.

Phthalate standards (used to enrich the molecular network) clustered within a network of 11 nodes (Fig. 7A), whereby MS2LDA and manual inspection analysis, using fragment search by mzCloud (www.mzcloud.org), led to the putative identification of phthalic acid-related chemistries, as per the motifs M2M_260 and M2M_191. The fragments 167.0325, 149.0225, and 121.0275 *m/z* suggest a phthalic acid-related core in five nodes within the network, as well as three additional single nodes (Fig. 7A). The assigned chemical class to the network (through ClassyFire) was benzene and substituted derivatives. Likewise, a group of three phenyl pentane derivatives was putatively annotated, two as carboxylic acids (5-phenylvaleric acids analogs) and one as a ketone, and all with double bonds. NAP consensus structures (within the top 10) of these compounds are illustrated in Fig. 7B and correlated with the predicted neutral losses and fragments (heuristic predictions) by MS2LDA and mzCloud.

**FIG 8:**
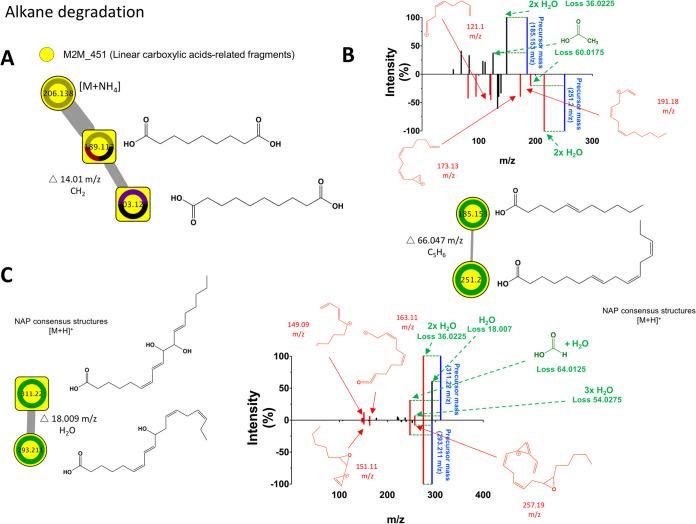
NAP/MS2LDA/mzCloud-driven metabolite annotation of selected chemistries linked to alkane degradation. (A) Identification of nonanedioic and decanedioic acids using analytical standards and annotation of M2M_451 as linear carboxylic acid-related fragments. (B) Putative identification of two polyunsaturated linear carboxylic acids. (C) Putative identification of two hydroxylated polyunsaturated linear carboxylic acids. Chemical structures drawn here are the top-ranked consensus candidates predicted by NAP. Neutral losses are depicted in green and fragments in red. Fragments drawn denote the heuristic fragmentation predictions by mzCloud (www.mzcloud.org).

Metabolites linked to alkane degradation were also found by spectral matching (and validated by analytical standards) and included two dicarboxylic acids, nonanedioic acid (C_9_H_16_O_4_) and decanedioic acid (C_10_H_18_O_4_) (24) (Fig. 8A; see also Table S3). Similarly, linear polyunsaturated fatty acids were putatively annotated with the help of MS2LDA and NAP and through manual inspection of neutral losses and fragments. Figure 8B shows the annotation of undecenoic acid (C_11_H_20_O_2_) and hexadecatrienoic acid (C_16_H_26_O_2_). The difference between both nodes is 66.047 *m/z* and is likely due to the fragment C_5_H_6_ containing two double bonds. Also, heuristic predicted fragments of 251.2 *m/z* suggest the presence of three double bonds that tightly correlate with the NAP consensus structure of a hexadecatrienoic acid derivative. However, the exact positions of unsaturations could not be determined. Noteworthy, polyunsaturated fatty acids have been reported in seawater contaminated with oil (25) and oil plumes (26), presumably linked to hydrocarbon degradation by native bacteria. Moreover, two additional hydroxylated C_18_ polyunsaturated fatty acids were putatively annotated (Fig. 8C). In addition to neutral losses of 36.0225 (double H_2_O loss) and 18.007 Da (H_2_O loss), 64.0125 (CHOOH loss)-Da and 54.0275 (triple H_2_O loss)-Da losses suggest the presence of a mono- and dihydroxylated polyunsaturated C_18_ fatty acid that correlates with NAP consensus structures (within the top five). The positions of the unsaturations could not be determined.

10.1128/mSystems.00824-20.9 TABLE S3 Analytical standards used for metabolite characterization or molecular network enrichment. Download Table S3, DOCX file, 0.07 MB. Copyright © 2020 Moreno-Ulloa et al. 2020 Moreno-Ulloa et al. This content is distributed under the terms of the Creative Commons Attribution 4.0 International license.

### Secondary metabolites and associated bacterial genera.

Metabolic evidence of phyla *Proteobacteria* and *Actinobacteria*, which correlated with 16S rRNA data (Fig. 4), was identified. Putative annotation of the carotenoid canthaxanthin was confirmed by spectral match (Fig. S3) and manual inspection against METLIN database. Canthaxanthin is produced by the genera *Bradyrhizobium* (class *Alphaproteobacteria*), *Gordonia* (class *Acidobacteria*), and *Dietzia* (class *Actinobacteria*) (27, 28). Likewise, two sphinganines (C_17_ and C_19_) were putatively annotated (see Fig. S4A and B). These compounds have been linked to the genus *Novosphingobium* (class *Alphaproteobacteria*) (29) and correlate with 16S rRNA findings. In addition, evidence of the genus *Streptomyces* (class *Actinobacteria*) was also provided. That is, by using a tetracycline standard to enrich the network, a 10-node cluster was putatively annotated as tetracycline-related chemistries (see Fig. S5). NAP also predicted a phenylpropanoid and polyketide superclass in this cluster that corresponds to the chemical classification of this well-known antibiotic produced by various *Streptomyces* strains (30).

## DISCUSSION

Marine microbes are capable of degrading petroleum derivatives through metabolic pathways that degrade specific hydrocarbons (31–33). Previous studies have focused on the environmental impact of oil spills on microbial communities and changes in taxonomic profiles over time (2, 5, 7, 8, 25, 26, 34–37). Metagenomic amplicon sequencing (e.g., 16S rRNA) and targeted metabolomics (e.g., gas chromatography) have contributed useful and substantial information to the field of microbial degradation of hydrocarbons. However, most studies have focused on microbes inhabiting the sea surface or shallow waters, while microbes inhabiting deep-sea sediments (>1,000 m below the sea surface) remain largely unexplored. The goals of this study were to obtain ODB from deep-sea sediments and profile the taxonomy and metabolism of the microbial community associated with the degradation of hydrocarbons. To achieve this, we used amplicon sequencing, untargeted metabolomic analyses, and chemoinformatics.

In agreement with previous and comprehensive reports on the GM, the findings from this study suggest a predominant abundance of *Proteobacteria* in environmental samples (7, 34, 36, 37). In one of the most comprehensive studies performed in the GM to date, where more than 700 marine sediment samples (water depth range, 16 to 2,293 m) from 29 sites across regions of the northern and southern GM were analyzed, Overholt and coworkers (37) suggested a consistent seafloor microbiome predominantly inhabited by *Proteobacteria*. Consistent with the findings from the present study, Overholt and coworkers (37) demonstrated that the *Nitrospira* genus was only present in deep-sea sediments of the GM. Although sediments A7 and B18 were collected from different locations, nearly half of the families were codetected in both sediments, with *Woeseiaceae* and *Kiloniellaceae* being the top two most abundant families. *Woeseiaceae* is one of the most abundant bacterial family in marine sediments (38).

Changes in microbial communities have been reported in hydrocarbon-contaminated environments. For instance, a higher abundance of *Proteobacteria* was found in sediments exposed to the oil spill in the DWH disaster (7). Accordingly, findings from this study support a shift in the bacterial community in sediments following culture with hydrocarbons (API 40 petroleum) toward a more similar taxonomic profile between samples collected from different locations. For instance, the cooccurrence of families among A7 and B18 increased from 46.15 to 95.5% after API 40 incubation. The ODB associated with the genera *Pseudomonas*, *Staphylococcus*, *Dietzia*, *Bacillus*, and *Vibrio* increased after 28 days in culture. Despite differences in the abundance of ODB, the global genera profile was similar between A7 and B18 after 28 days.

It must be noted however that *in vitro* simulations present some limitations. For example, A7 and B18 microbes grew during this culture period (according to oxygen consumption analysis), although a lower number of total families were detected compared to initial native conditions. These findings suggest that the *in vitro* conditions established in this study did not support growth of all microbes; however, as expected the microbes that did survive were associated with hydrocarbon-degrading properties (32). Similar observations were made in a previous study simulating deep-sea environments (pressure, 0.1 and 30 MPa; temperature, 5 and 20°C) where the microbial community structure remained similar at the phylum level but differed at the taxon level (39).

Oxygen concentration was not measured in environmental sediments when collected (core of 0 to 10 cm below seafloor); however, previous studies suggest oxygen penetration up to 15 cm below the seafloor in GM deep-sea sediments (>1,000 m) (5). Furthermore, metagenome shotgun sequencing predicted aerobic and anaerobic processes in the surface (0 to 1 cm below seafloor) of oil-contaminated deep-sea sediments of the GM (7). The culturing method in this study involved sealed bottles (finite oxygen supply); therefore, it can be assumed that anoxic and oxic conditions may have led to both aerobic (early times) and anaerobic (late times) microbial metabolic pathways. Subsequently, the culture conditions in this study did not fully mimic those found in a deep-sea sediments niche. For example, achieving the temperature, and more importantly the pressure of deep-sea environments, was beyond the capabilities of a general laboratory. Importantly, one of the main goals of the present study was to utilize accessible laboratory conditions for the culture of deep-sea microbes that could degrade hydrocarbons and facilitate chemical and taxonomic profiling.

Nitrogen availability has been reported as a limiting factor for the degradation of hydrocarbons in marine sediments (40). Higher nitrate consumption has been observed in oil-contaminated deep-sea sediments (∼1500 m deep) close to the DHW wellhead compared to uncontaminated sediments (41). The effects of dissolved nitrogen (in the form of NO_3_^–^ at 0, 0.0004, 0.004, 0.04, and 4 g liter^−1^) on microbial growth during 28 days in culture with API 40 were investigated. Consistent with previous studies, higher microbial growth was observed with the highest concentration of nitrate (4 g liter^−1^), while no microbial growth occurred at concentrations of <0.04 g liter^−1^.

Several studies have predicted the capacity of marine microbes to degrade hydrocarbons based on the presence of particular bacterial communities (i.e., ODB) (7, 31). However, these predictions require validation by complementary approaches such as metabolomics. Therefore, a nontargeted and nonclassical metabolomic approach was performed in this study to gain insights into the potential of deep-sea microbes to degrade hydrocarbons and to improve the current knowledge of the metabolic machinery in these microbes. The metabolome of marine microbes currently available contains molecules with a mass range of 147.044 to 882.687 Da with low cooccurrence or overlapping between microbial samples, as well as a relatively low spectral match (<2%) against public spectral libraries (level 2 annotations according to the 2007 metabolomics standards initiative [42]).

It must be noted that Bargiela et al. (43) used HPLC-MS (without MS/MS) to fingerprint the chemical profile associated with microbial metabolism in chronically petroleum-polluted coastline sediments. Features up to ∼1,870 *m/z* (positive ionization mode) were reported with only accurate mass ([M+H]^+^) shown; however, putative annotation of such compounds could not be achieved due to a lack of MS/MS data and advanced dereplication tools (43). To overcome the low dereplication rate observed in the present study, *in silico* structure prediction (by NAP) was applied to obtain *in silico* fragmentation-based metabolite annotation (as reported by others [15, 19, 23]). This type of analysis enabled most of the nodes (or metabolites) to be annotated (by a consensus candidate structure) within the global molecular network and assigned a chemical ontology to each annotated metabolite using ClassyFire (44).

Compared to other studies targeting specific metabolic intermediates of hydrocarbon degradation by microbes (8, 45, 46), the present study highlights the diverse chemical profile produced by deep-sea microbes when cultured with hydrocarbons. It is important to clarify that the metabolome analyzed could contain molecules derived from microbial cell walls. Therefore, the metabolome was first described in global terms followed by a focused approach. For example, this analysis focused on specific subnetworks, or clusters of interest (e.g., containing standards or confidently annotated metabolites), guided by the ClassyFire chemical classification (44) that inferred the presence of metabolites associated with hydrocarbon degradation. Notably, and despite similar taxonomic profiles between A7 and B18 following API 40 incubation, a low cooccurrence was observed at the metabolite level and could suggest marked differences in metabolism. A similar observation was made following incubation of SO with A7 and B18. Unfortunately, a taxonomic profile was not obtained for samples incubated with SO. The aim of this study was to culture with SO and use known metabolites to facilitate profiling (by inference) of deep-sea microbe metabolites derived from API 40 degradation.

The most abundant chemical superclass identified was lipids and lipid-like molecules, and included typical hydrocarbon derivatives (e.g., linear carboxylic acids, alcohols, etc.,) similar to those reported as petroleum products by microbial metabolism (31). Furthermore, two saturated dicarboxylic acids derived from alkane degradation, nonanedioic and decanedioic acid (Fig. 8A; see also Table S3) (24) (level 1 annotation according to the 2007 metabolomics standards initiative [42]), were identified in this study using analytical standards. MS2LDA was also combined with mzCloud to predict that most of the metabolites generated by A7 and B18 were hydroxylated and carboxylated.

The multiapproach used in the present study to dereplicate metabolites also suggested the presence of C_16_ and C_18_ polyunsaturated fatty acids (Fig. 8B) and hydroxylated C_18_ fatty acids (Fig. 8C). Saturated and polyunsaturated fatty acids have been reported in seawater contaminated with oil (25) and oil plumes (26) and are presumably linked to hydrocarbon degradation by native bacteria. Although both saturated and polyunsaturated fatty acids have been described as intermediates of alkane microbial degradation (47), less is known about the role of hydroxylated polyunsaturated entities. Interestingly, alkane hydroxylases from the family of cytochrome P450 enzymes have been described in hydrocarbon-degrading bacteria (48). In this study, PICRUSt revealed an increased abundance of sequences linked to cytochrome P450 (level 3, KEGG orthology) in samples grown with API 40 compared to environmental samples. These findings also correlate with the metabolomic outcomes in this study. Similarly, PICRUSt projections suggested an increase in linolenic acid metabolism (fatty acid polyunsaturated, three unsaturations) that correlated with putative annotated fatty acids containing three double bonds.

Compelling evidence was provided by this study regarding the metabolism of alkanes by marine microbes involving fatty acid intermediate metabolites (see Table S4). The different energy sources (API 40 and SO) used to culture deep-sea microbes enabled the synthesis of saturated and polyunsaturated fatty acids to be linked to a particular substrate. For instance, dicarboxylic acids were identified when microbes were cultured with either API 40 (a complex unknown mixture of hydrocarbons) or SO (containing only hexadecane, naphthalene, toluene, and benzene), while polyunsaturated fatty acids were only detected in microbes cultured with SO. These findings reveal the possible substrates that could be utilized for production of such fatty acid metabolites, and according to published studies (49, 50), we infer that hexadecane is the most likely substrate used by microbes belonging to the genus *Pseudomonas* (that were highly abundant in API 40-enriched samples). However, further studies must be performed using isolated bacteria strains and are currently ongoing.

10.1128/mSystems.00824-20.10 TABLE S4 Putative annotated metabolites by NAP and MS2LDA tools and manual inspection. Download Table S4, DOCX file, 0.1 MB. Copyright © 2020 Moreno-Ulloa et al. 2020 Moreno-Ulloa et al. This content is distributed under the terms of the Creative Commons Attribution 4.0 International license.

Prediction of metabolic potential using PICRUSt also suggested increased representation of aromatic degradation pathways in API 40-enriched samples. Using analytical standards of various phthalates (see Table S3) containing substructurally a phthalic acid core (i.e., benzene 1,2-dicarboxylic acid) that resembles a metabolic intermediate of polycyclic aromatics (33), the presence of phthalic acid derivatives was inferred in samples cultured with SO. The nontargeted approach analyzing MS/MS data indicated that various metabolites with a phthalic acid core were linked to naphthalene degradation. Metabolites with a phthalic core were detected in samples cultured with SO wherein naphthalene was one of the hydrocarbons present (and microbial metabolism of naphthalene could lead to the generation of phthalic acid) (33). However, due to the relatively high mass and lack of confident substructure matches against mzCloud, compounds within this cluster of phthalic acid derivatives could not be annotated, even with the assistance of informatics tools. Phthalic anhydride (see Fig. S2) was also detected and is associated with unusual anaerobic microbial degradation of o-phthalates via the benzoyl-CoA degradation pathway (51). An extensive dereplication of the data may reveal other novel metabolites linked to microbial utilization of aromatic hydrocarbons. However, this was deemed beyond the study scope due to the complexity and time required.

10.1128/mSystems.00824-20.3 FIG S2 MS2 spectrum of phthalic anhydride. Fragments were putatively annotated using heuristic predictions by mzCloud. Download FIG S2, JPG file, 0.3 MB. Copyright © 2020 Moreno-Ulloa et al. 2020 Moreno-Ulloa et al. This content is distributed under the terms of the Creative Commons Attribution 4.0 International license.

10.1128/mSystems.00824-20.4 FIG S3 Mirror plot of MS2 library and query spectra. Fragments were putatively annotated using METLIN. Precursor ion, 565.4099 *m/z.* Exact mass, 565.404. Mass error, 10.4 ppm. Plots were retrieved from GNPS. Download FIG S3, JPG file, 0.2 MB. Copyright © 2020 Moreno-Ulloa et al. 2020 Moreno-Ulloa et al. This content is distributed under the terms of the Creative Commons Attribution 4.0 International license.

10.1128/mSystems.00824-20.5 FIG S4 (A) Mirror plot of MS2 library and query spectra. Spectral match of C_17_ sphinganine against GNPS library. Fragments were putatively annotated using mzCloud Heuristic predictions. Precursor ion, 288.29 *m/z.* Exact mass, 288.2903. Mass error, −1.04 ppm. There is a neutral H_2_O loss (18.0125) from 288.29 to 270.2775. Plots were retrieved from GNPS. B) Sphinganine-containing cluster. Putative annotation of C_17_ and C_19_ sphinganines. Structures were retrieved from NAP consensus candidates (first). Precursor ion, 288.29 *m/z;* exact mass, 288.2903 *m/z*; mass error, −1.04 ppm. Precursor ion, 316.3219 *m/z;* exact mass, 316.3216 *m/z*; mass error, 0.94 ppm. Plots were retrieved from GNPS. Download FIG S4, JPG file, 0.2 MB. Copyright © 2020 Moreno-Ulloa et al. 2020 Moreno-Ulloa et al. This content is distributed under the terms of the Creative Commons Attribution 4.0 International license.

10.1128/mSystems.00824-20.6 FIG S5 Cluster with tetracycline-related metabolites. Putative annotation of nodes containing a tetracycline core or alike substructure. Node attached to tetracycline standard (STD) was plotted as a mirror plot using MS2 spectra. Various fragments were shared among unknown metabolite 444.698 *m/z* and tetracycline STD suggesting a related structure. Fragment 410.1225 *m/z* (M2M_14) was assigned to a tetracycline-core (red substructure) using Heuristic fragmentation prediction by mzCloud (www.mzcloud.org). Download FIG S5, JPG file, 0.4 MB. Copyright © 2020 Moreno-Ulloa et al. 2020 Moreno-Ulloa et al. This content is distributed under the terms of the Creative Commons Attribution 4.0 International license.

Three phenyl pentenoids, including two carboxylic acids and one ketone, were also annotated in this study (see Table S4). Similar phenyl valeric acids, but with aromatic hydroxylations (i.e., ortho-dihydroxy benzene substructure), have been reported as gut microbiota-derived metabolites linked to flavonoid degradation via ring opening (52). In the present study, the three phenyl pentenoids were detected following microbial growth with SO. Thus, the most likely substrate is naphthalene (due to the chemical structure), although this assumption requires further interrogation. In agreement with these findings (Fig. 5A and B), Neethu et al. (25) also showed an increase in the representation of genes coding for transcription factors in oil sediment and seawater, suggesting an increase in microbial molecular activity. It must also be noted that the accuracy of functional predictions by PICRUSt may be limited. That is, predictions may be less accurate in complex and less well-studied environments, like deep-sea sediments, where reference genomic information is minimal (21). However, PICRUSt predictions in this study were further supported by metabolomics that increased confidence in the metabolic profiles identified (e.g., aromatic and alkane metabolism) following growth of A7 and B18 microbes with hydrocarbons.

**FIG 9:**
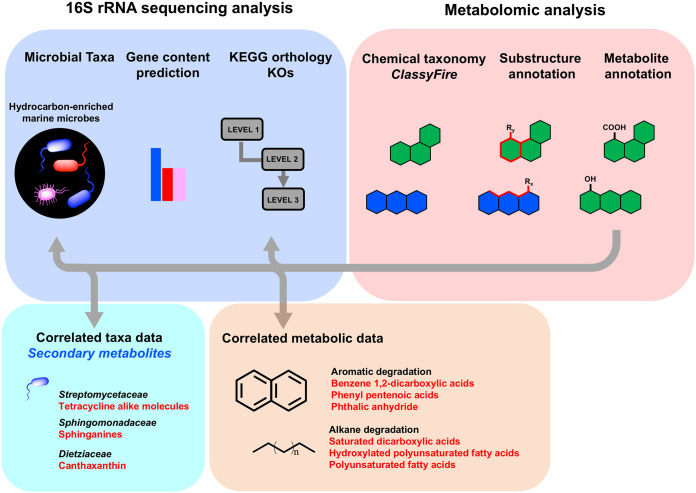
Summary of methodology and findings.

Despite advances in the taxonomic profiles reported for the GM, there is limited information that describes the metabolic machinery of marine microbes with the capacity to produce secondary metabolites when hydrocarbons are the sole energy source. Metabolomics has the potential to profile bacterial taxa by identifying secondary metabolites linked to specific genera or species. Nuclear magnetic resonance spectroscopy and mass spectrometry have been used to differentiate bacteria in infected lungs (53), identify bacterial species *in vitro* (54, 55), and distinguish Pseudomonas aeruginosa clone TB strains from cystic fibrosis airways (56). Therefore, metabolomic and 16S rRNA data sets were compared in this study to provide compelling evidence about microbial communities in API 40-enriched samples. Both approaches provided evidence of genera *Dietzia* (family *Dietziaceae*), *Novosphingobium* (family *Sphingomonadaceae*), and *Streptomyces* (family *Streptomycetaceae*) with ODB capabilities that was supported by previous reports of associations between these genera and ODB (20, 28). Hence, the combined 16S rRNA sequencing and metabolomics approach allowed confident characterization of the metabolic state and taxa of microbes grown with hydrocarbons (Fig. 9).

In conclusion, this study provides previously unreported evidence of deep-sea microbes thriving in the presence of toxic hydrocarbons, and reveals the chemical repertoire of metabolites associated with microbial degradation of hydrocarbons. This study also highlights the suitability of untargeted metabolomics and comprehensive chemoinformatics to evaluate the capacity of microbes to use hydrocarbons as an energy source.

## MATERIALS AND METHODS

### Sample collection.

Two superficial sediment samples (0 to 10 cm) from water depths of 1265 m (designated as B18 [23°54′57.6″N, 86°47′28.8″W]) and 3500 m (designated as A7 [24°57′37.74″N, 90°0′52.5″W]) were collected using a Reineck box core (50 × 50 cm) in August 2015 during the GM oceanographic campaign XIXIMI-4 (Fig. 1). Samples were collected and stored in sterile cap tubes of 10 cm^3^ at −20°C while onboard and then at −80°C in the laboratory until processing.

### Culture of marine sediments.

The methodology followed in this study is illustrated in Fig. 2. For analyses, two general steps were followed: (i) A7 and B18 deep-sea sediments from the sterile cap tubes (for consistency, these are referred to here as “environmental sediments”) were used for 16S rRNA amplicon sequencing analysis without any further incubations or treatments, and (ii) A7 and B18 environmental samples were manipulated to obtain oil-degrading bacteria (ODB).

For culture in the presence of hydrocarbons (step ii), sediments (one full tube of 10 cm^3^) were first cultured with a mineral medium containing inorganic salts (4 g liter^−1^ NaNO_3_, 2 g liter^−1^ K_2_HPO_4_, 1 g liter^−1^ MgSO_4_⋅7H_2_O, 1 g liter^−1^ KCl, 0.02 g liter^−1^ FeSO_4_⋅7H_2_O) and trace elements (0.26 g liter^−1^ H_3_BO_3_, 0.50 g liter^−1^ CuSO_4_⋅5H_2_O, 0.50 g liter^−1^ MnSO_4_⋅H_2_O, 0.06 g liter^−1^ MoNa_2_O_4_⋅H_2_O, 0.70 g liter^−1^ ZnSO_4_⋅7H_2_O, 0.0185 g liter^−1^ CoCl_2_) dissolved in 50 ml of seawater (filtered by a 0.2-μm membrane) at pH 7 ± 0.2, as described by Abalos et al. (57) with modifications made to concentration and salt type. As the sole source of carbon, medium was supplemented with crude oil American Petroleum Institute 40 (API 40) gravity (0.4% [vol/vol]). Cultures were maintained under constant agitation at 200 rpm (MaxQ 4000 benchtop orbital shaker; Thermo Fisher Scientific, Waltham, MA) in darkness for 4 weeks at 19 ± 1°C. After this enrichment with ODB, the samples were filtered using sterile cotton gauzes to remove sediment particles and then divided into 1-ml aliquots (containing 0.5% [vol/vol] glycerol) for long-term storage at −80°C. Microbial growth was estimated using the most-probable-number (MPN) method (58). Next, ∼5.9 × 10^4^ MPN were cultured with API 40 for four additional weeks to allow ODB enrichment and removal of residual glycerol. Samples were then centrifuged at 4,500 rpm for 10 min with the pellet retained and washed once with mineral medium, and then resuspended in fresh mineral medium. Finally, ∼5.9 × 10^4^ MPN (free of sediments and traces of glycerol) were transferred into OxiTop flasks (WTW, Weinheim, Germany) containing mineral medium (total volume 43.226 ml) and either API 40 (0.4% [vol/vol]) or synthetic oil (SO; 87 μl of a mixture, on a molar basis, of toluene [72.15%], benzene [8.27%], naphthalene [11.33%], and hexadecane [8.25%]) as the sole source of energy. After the OxiTop flasks were filled, a carbon dioxide trap was fixed in each flask containing three pellets of KOH. Bottles were then capped with OxiTop heads and subjected to magnetic stirring (200 rpm) to ensure homogeneity at 19 ± 1°C for 4 weeks (referred to as “final times”). Data collection started immediately. Samples treated as described above, but without incubation, are referred to as “initial times.” Also, a set of control flasks was used to ensure the reliability of the culturing method: (i) flasks containing mineral medium and either API 40 oil or SO without inoculum and (ii) flasks containing mineral medium and inoculum without API 40 or SO. The OxiTop system enables measurement of oxygen consumption rate indirectly evaluating substrate utilization by microbes and aerobic degradation (since oxygen inside the bottle is in equilibrium with that of the environment before closing the cap) (59–61). However, after oxygen depletion, anaerobic degradation could occur. The experiments were performed separately in triplicate for amplicon sequencing and metabolomic analysis.

### DNA extraction.

DNA was extracted from environmental sediments (without further processing) and from samples grown for 28 days with hydrocarbons. The total DNA was extracted by a PowerMarx soil (MoBio, Carlsbad, CA) DNA isolation kit according to the manufacturer’s instructions. The concentration and purity of extracted DNA were determined using a NanoDrop Lite spectrophotometer (Thermo Fisher Scientific, Waltham, MA). Due to limited DNA yield from environmental sediments, triplicates were pooled for DNA extraction and further processing.

### 16S rRNA amplicon sequencing.

16S rRNA sequencing focused on seven hypervariable regions (V2 to V4 and V6 to V9) generated from 1 μg of purified DNA, implemented in an Ion Personal Genome Machine (PGM) system (Thermo Fisher Scientific). Sequencing was carried out according to an Ion 16S Metagenomics kit (A26216; manual MAN0010799; Thermo Fisher Scientific).

Sequences 100 to 400 bp long with a quality of ≥25 were selected for analysis. Filtered reads were used to remove chimeric reads using VSEARCH (62) using the Gold database (http://drive5.com/uchime/uchime_download.html). The OTUs were generated by the UCLUST method implemented in QIIME version 1.9 (63), using the close reference model implemented at 97% similarity with the Silva database release 132 (64). The alpha-diversity metrics were implemented using the Shannon index. Detected bacteria were plotted (i.e., heatmaps) as relative abundance showing the most abundant families in API 40-enriched and environmental samples using Phyloseq v1.28 in R (65). Functional diversity was predicted using 16S rRNA data and PICRUST v1.1.4, with the Kyoto Encyclopedia of Genes and Genome (KEGG) database (21), and results were visualized (using the KEGG Orthology [KO] classification scheme) and analyzed using the software package STAMP v2.0 (66). Environmental and petroleum-enriched samples from the same type of sediment were compared by the ‘‘two samples’’ analysis function within STAMP software using the following statistical parameters: statistical test, Fisher exact test; type, two sided; CI method, Newcombe-Wilson; and multiple test correction, Benjamini-Hochberg FDR, according to the recommended statistical practices of the STAMP user’s guide.

### Metabolite extraction.

Entire flasks of initial and final time samples were transferred to Nalgene Teflon Oak Ridge tubes (Thermo Fisher Scientific) before a solvent mixture of dichloromethane and ethyl acetate (50:50 [vol/vol]) was added to extract the metabolites. Tubes were mixed using gentle and slow movements before being centrifuged at 5,000 rpm for 3 min. Organic solvents were recovered, dried over Na_2_SO_4_, concentrated in a vacuum rotary evaporator, and finally dried under a gentle nitrogen stream. Samples were stored at −20°C until processing.

### LC-MS/MS data acquisition.

Dried extracts were reconstituted in 5% acetonitrile (ACN) in water with 0.1% formic acid and then centrifuged at 14,000 rpm for 10 min at 4°C. The particle-free supernatant was recovered and analyzed using an Eksigent nanoLC 400 system (AB Sciex, Foster City, CA) with a HALO phenyl-hexyl column (0.5 × 50 mm, 2.7 μm, 90-Å pore size; Eksigent; AB Sciex). Metabolites were separated using gradient elution with 0.1% formic acid in water (A) and 0.1% formic acid in ACN (B) as mobile phases at a constant flow rate of 5 μl/min. The gradient started at 5% B for 1 min, followed by a stepped increased to 100% B over 26 min and held constant for 4 min. Solvent composition was then returned to 5% B over 0.1 min. To ensure column reequilibration, a 4-min pre-run with 5% B was applied between samples. A blank sample (1 μl of buffers A and B at a 95:5 ratio) was run between experimental sample injections to minimize potential carryover. The eluate from LC was delivered directly to the TurboV source of a TripleTOF 5600+ mass spectrometer (AB Sciex) using electrospray ionization (ESI) under positive mode. ESI source conditions were set as follows: IonSpray voltage floating, −5,500 V; source temperature, 350°C; curtain gas, 20 lb/in^2^; ion source gases 1 and 2 were set to 40 and 45 lb/in^2^; and declustering potential, −100 V. Data were acquired using information-dependent acquisition (IDA) with high-sensitivity mode selected, automatically switching between full-scan MS and MS/MS. The accumulation time for TOF MS was 0.25 s/spectra over the *m/z* range 100 to 900 Da, and for the MS/MS scan it was 0.05 s/spectra over the *m/z* 50 to 900 Da. The IDA settings were as follows: charge state, +1 to +2; intensity, 125 cps; exclude isotopes within 6 Da; mass tolerance, 50 mDa; and a maximum number of candidate ions 20. Under IDA settings, the “exclude former target ions” was set as 15 s after two occurrences and “dynamic background subtract” was selected. The manufacturer rolling collision energy (CE) option was used based on the size and charge of the precursor ion using the formula CE = *m/z* × 0.0575 + 9. The instrument was automatically calibrated by the batch mode using appropriate positive TOF MS and MS/MS calibration solutions before sample injection and after injection of two samples (<3.5 working hours) to ensure a mass accuracy of <5 ppm for both MS and MS/MS data. Instrument performance was monitored during data acquisition by including QC samples (pooled samples of equal volume) every four experimental samples. Data acquisition of experimental samples was also randomized.

### LC-MS/MS data processing and analysis.

LC-MS/MS data sets were processed using MZmine (67). Features detected in initial times and common contaminants (i.e., a list of features detected in a run with mobile phase used for sample preparation) were subtracted from each final time data set to retain the features exclusively associated with hydrocarbon metabolism after 28 days of culture. To profile the chemistry of microorganisms with hydrocarbon-degrading capabilities, MZmine-processed LC-MS data sets from A7 and B18 grown with both API 40 and SO were submitted to the Global Natural Products Social Molecular Networking web platform (GNPS; https://gnps.ucsd.edu) (16). To further characterize the chemistries of B18 and A7, the NAP tool (15) was used to propagate structural annotations by *in silico* predictions and automatically classify the molecular networks as per the most predominant chemical superclass (68) using the ClassyFire tool (44). The most abundant superclass within each cluster represents the entire cluster (68). As a complementary approach to NAP, the MS2LDA computational tool was also used to discover cooccurring fragments and neutral losses (named M2M) within the MS2 data (using the same .mgf file as in GNPS and NAP) to provide chemical information at the substructure level (17). A detailed procedure is described in Text S1 in the supplemental material.

10.1128/mSystems.00824-20.1 TEXT S1 LC-MS/MS data processing using MZmine. Download Text S1, DOCX file, 0.03 MB. Copyright © 2020 Moreno-Ulloa et al. 2020 Moreno-Ulloa et al. This content is distributed under the terms of the Creative Commons Attribution 4.0 International license.

### Statistical analysis.

Bacterial growth experiments were performed in triplicate with data expressed as mean and SEM. To determine the growth rate among A7 and B18, exponential growth phases were fitted with a linear regression and slopes were compared by a two-tailed test (*P* value <0.05) using Prism, version 6.0. Using metabolomics, a minimum of 10-fold increase (triplicate mean of area under the curve) was used to qualitatively determine the appearance of metabolites after 28 days of microbial growth between samples at the initial and final times.

### Data availability.

Data available include the following: GNPS molecular network, https://proteomics2.ucsd.edu/ProteoSAFe/status.jsp?task=83e5ad0faf9a4d9e98c712f89ff89aab; NAP molecular networks, https://proteomics2.ucsd.edu/ProteoSAFe/status.jsp?task=0437a58d70014d26b7e359b5dbd51578; MS2LDA network, http://www.ms2lda.org/basicviz/view_mass2motifs/1107/; and 16S rRNA, NCBI SRA accession number PRJNA622289.
