# Supplementary figures and images for: Chemical Profiling Provides Insights into the Metabolic Machinery of Hydrocarbon-Degrading Deep-Sea Microbes
Source: mSystems. 2020 Nov 10;5(6):e00824-20. doi: 10.1128/mSystems.00824-20 (PMC7657597; doi:10.1128/mSystems.00824-20)

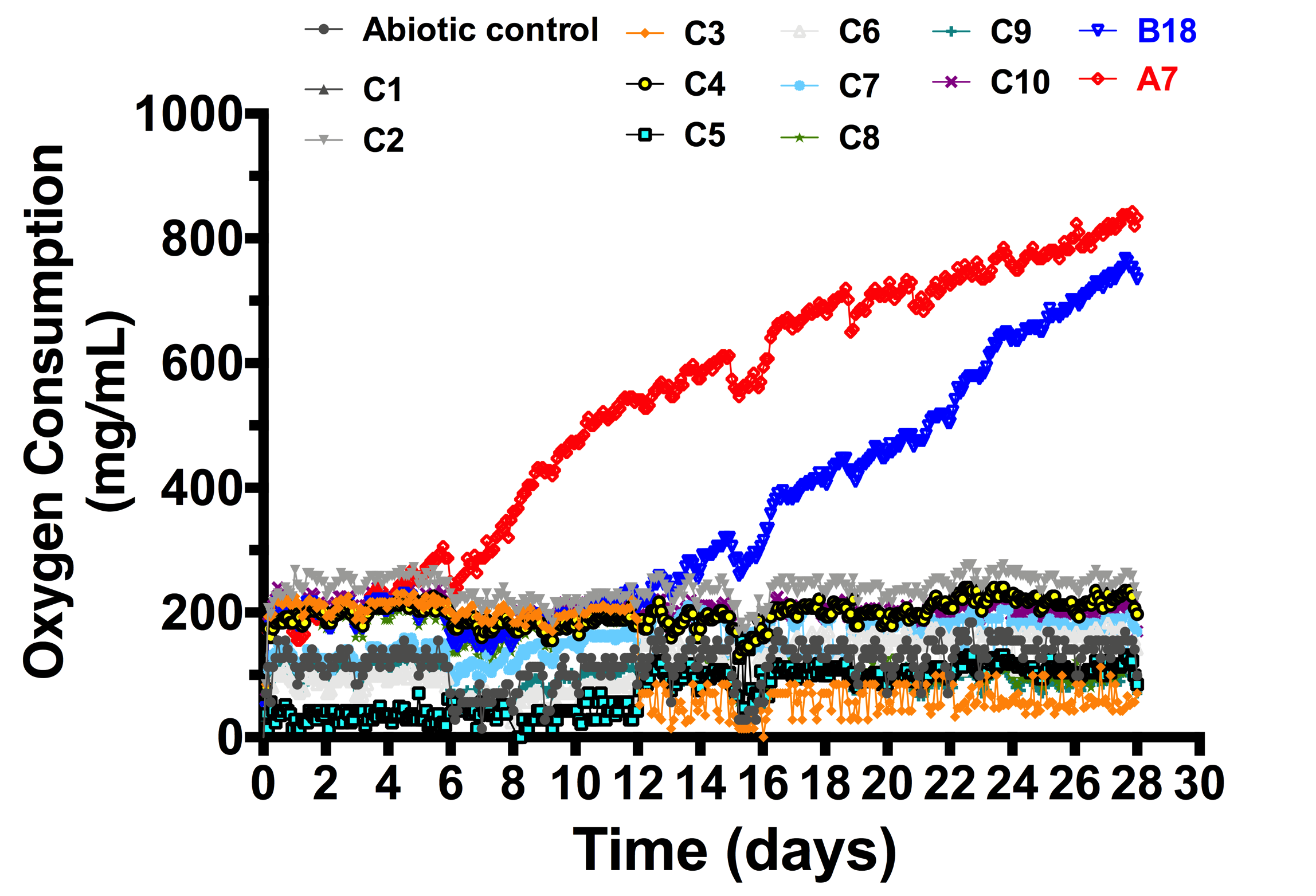

Supplement: FIG S1 [file mSystems.00824-20-sf001.tif]

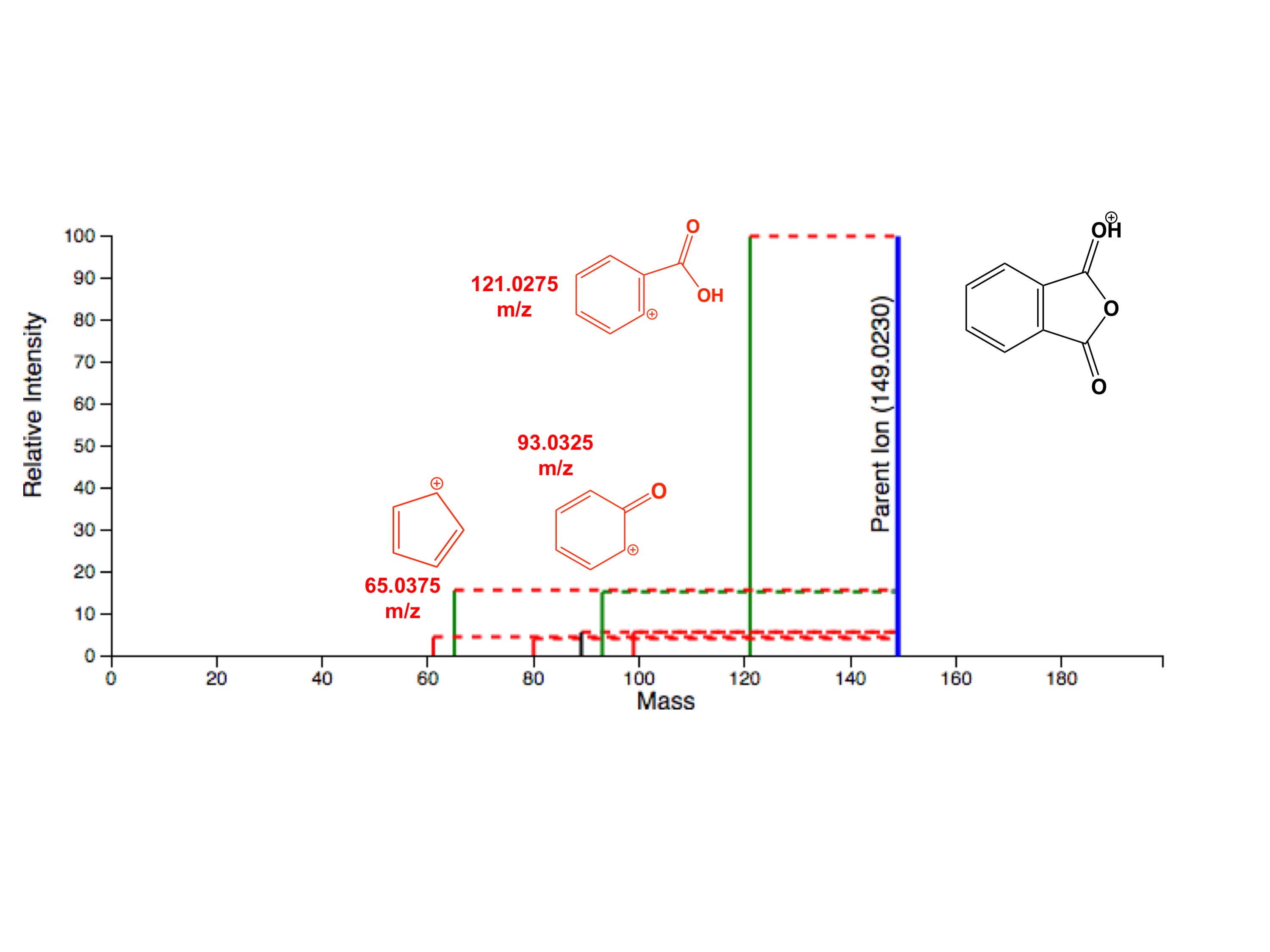

Supplement: FIG S2 [file mSystems.00824-20-sf002.jpg]

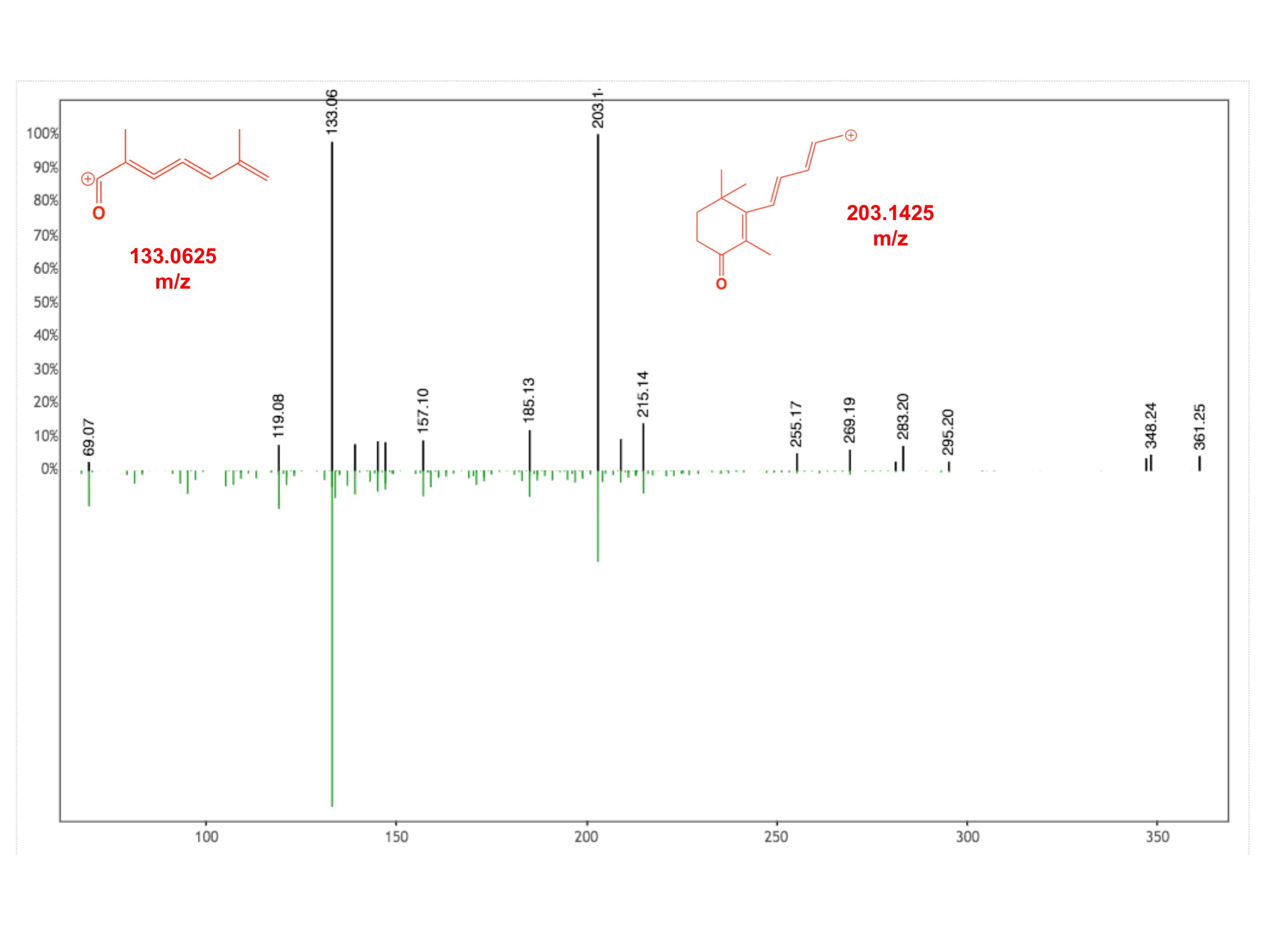

Supplement: FIG S3 [file mSystems.00824-20-sf003.jpg]

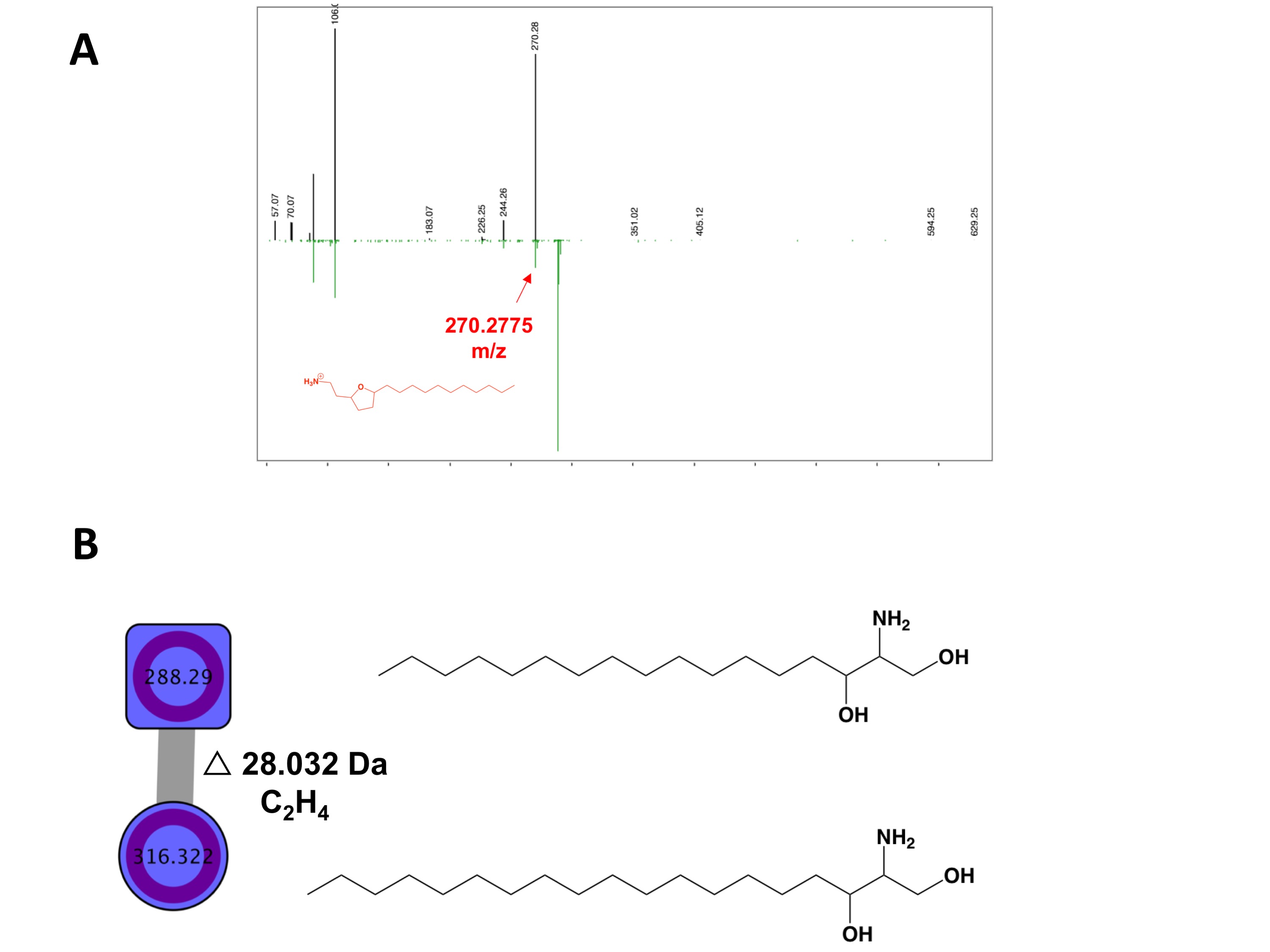

Supplement: FIG S4 [file mSystems.00824-20-sf004.jpg]

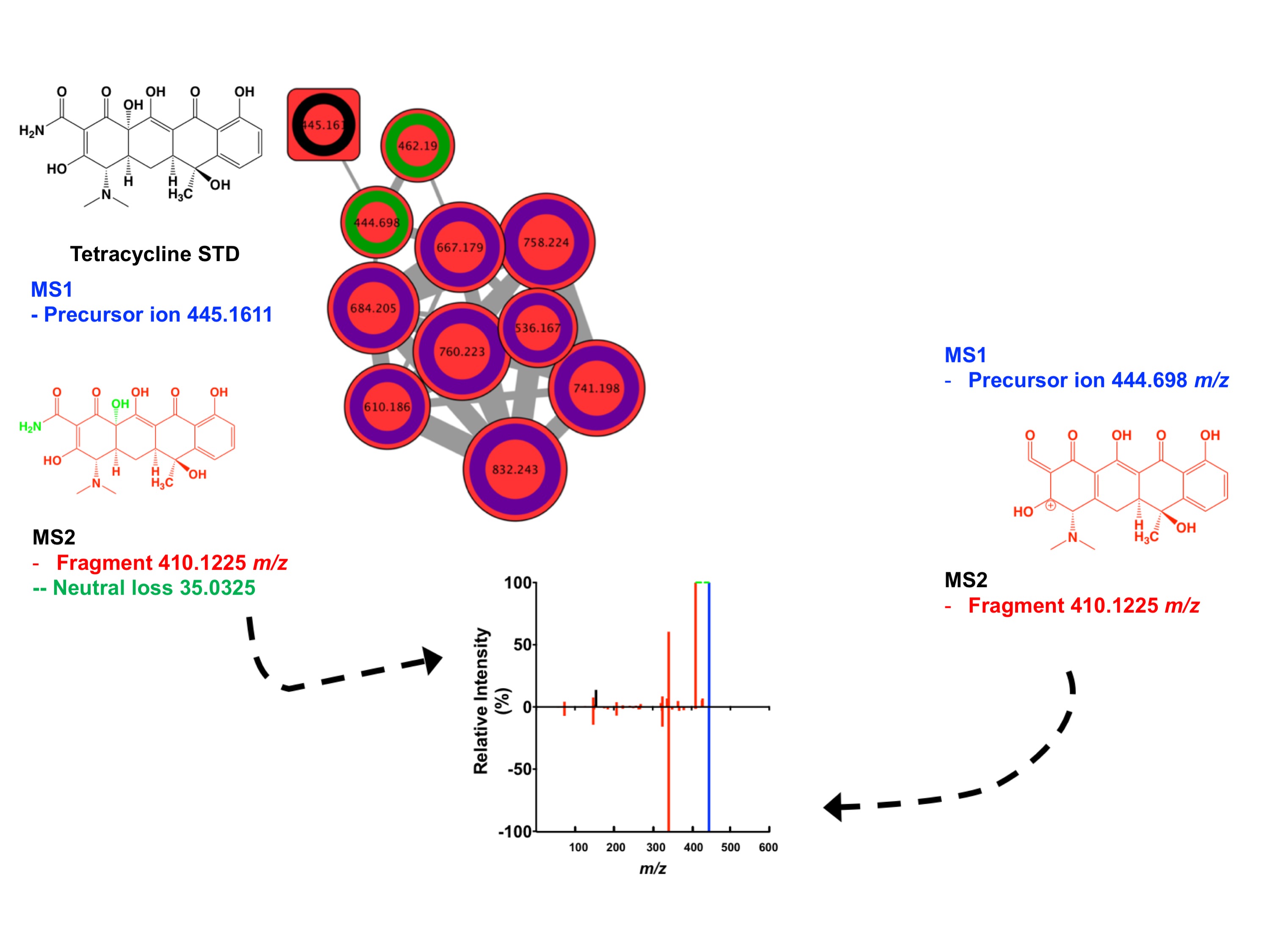

Supplement: FIG S5 [file mSystems.00824-20-sf005.jpg]
