# Supplementary material for: Chemical Profiling Provides Insights into the Metabolic Machinery of Hydrocarbon-Degrading Deep-Sea Microbes
Source: mSystems. 2020 Nov 10;5(6):e00824-20. doi: 10.1128/mSystems.00824-20 (PMC7657597; doi:10.1128/mSystems.00824-20)
Supplement: TABLE S3 [file mSystems.00824-20-st003.docx]

**Table S3. Authentic standards used for metabolite characterization or molecular network enrichment**

| Compound | Exact mass  [M+H]+ | Experimental  [M+H]+ | Mass accuracy (ppm) | Retention time (min) | Sample where detected |
| --- | --- | --- | --- | --- | --- |
| Azelaic acid | 189.1127 | 189.1120 | -3.7 | 7.9 | B18-API40 (NH_4_^+^ adduct), A7-SO, B18-SO |
| Sebacic acid | 203.1284 | 203.1280 | -1.9 | 9.1 | B18-API 40, A7-SO, B18-SO |
| Benzyl butyl phthalate | 313.144 | 313.144 | 0 | 21.4 | Standard used for network enrichment |
| Dibutyl phthalate | 279.16 | 279.1597 | 1.07 | 21.4 | Standard used for network enrichment |
| Dioctyl phthalate | 391.285 | 391.2849 | 0.2 | 26.1 | Standard used for network enrichment |
| Diethyl phthalate | 223.096 | 223.0971 | -4.9 | 15.5 | Standard used for network enrichment |
| Amoxicilin | 366.1124 | 366.1120 | -1.09 | 2.08 | Standard used for network enrichment |
| Levofloxacin | 362.1516 | 362.1510 | -1.6 | 10.07 | Standard used for network enrichment |
| Ceftriaxone | 555.0539 | 555.0540 | 0.18 | 15.5 | Standard used for network enrichment |
