# Supplementary material for: Chemical Profiling Provides Insights into the Metabolic Machinery of Hydrocarbon-Degrading Deep-Sea Microbes
Source: mSystems. 2020 Nov 10;5(6):e00824-20. doi: 10.1128/mSystems.00824-20 (PMC7657597; doi:10.1128/mSystems.00824-20)
Supplement: TABLE S4 [file mSystems.00824-20-st004.docx]

**Table S4. Putative annotated metabolites by NAP, MS2LDA tools and manual inspection.**

| Compound | Exact mass  [M+H]+ | Experimental  [M+H]+ | Mass accuracy (ppm) | Annotated by MS/MS | Associated degradation route |
| --- | --- | --- | --- | --- | --- |
|  | 173.0967 | 173.0959 | -4.6 | Yes | Aromatics |
|  | 191.1072 | 191.1063 | -4.7 | Yes | Aromatics |
|  | 177.0916 | 177.0916 | 0 | Yes | Aromatics |
|  | 185.1542 | 185.1534 | -4.3 | Yes | Alkanes |
|  | 251.2011 | 251.2001 | -3.9 | Yes | Alkanes |
|  | 311.2223 | 311.22 | -7.3 | Yes | Alkanes |
|  | 293.2117 | 293.2114 | -1.0 | Yes | Alkanes |
