# Supplementary material for: Chemical Profiling Provides Insights into the Metabolic Machinery of Hydrocarbon-Degrading Deep-Sea Microbes
Source: mSystems. 2020 Nov 10;5(6):e00824-20. doi: 10.1128/mSystems.00824-20 (PMC7657597; doi:10.1128/mSystems.00824-20)
Supplement: TEXT S1 [file mSystems.00824-20-s0001.docx]

***Supplementary File 1. LC- MS/MS data processing and analysis***

**Chemical profiling provides insights into the metabolic machinery of hydrocarbon-degrading deep-sea microbes**

Aldo Moreno-Ulloa^a,b^, Victoria Sicairos Diaz^b^, Javier A. Tejeda-Mora^a,b,c^, Marla I. Macias Contreras^b^, Fernando Díaz Castillo^b^, Abraham Guerrero^b,d^, Ricardo Gonzalez Sanchez^a,b^, Omar Mendoza-Porras^e^, Rafael Vazquez Duhalt^b,c^, and Alexei Licea-Navarro^a,b,#^

^a^ Departamento de Innovación Biomédica, Centro de Investigación Científica y de Educación Superior de Ensenada, Baja California (CICESE), México

^b^ Consorcio de Investigación del Golfo de México (CIGOM), CICESE, México

^c^ Departamento de Bionanotecnología, Centro de Nanociencias y Nanotecnología, Universidad Autónoma de México (UNAM), México

^d^CIAD/Mazatlán Unit for Aquaculture, AP. 711, Mazatlán Sinaloa, Mexico

^e^CSIRO Livestock and Aquaculture, Queensland Bioscience Precinct, 306 Carmody Rd, St Lucia, QLD, Australia

**Correspondence to:**

Alexei Licea-Navarro

[alicea@cicese.mx](mailto:alicea@cicese.mx)

**LC- MS/MS data processing and analysis**

Raw files (.wiff and wiff.scan) were converted to .mzXML using ProteoWizard (1) and processed with MZmine 2.34 (2). Centroided mass detection was done keeping the noise level at 1 and 0.01 for MS1 and MS2, respectively. Chromatograms were built using the ADAP algorithm (3) by inputting the following parameters: intensity threshold of 5.0, minimum highest intensity of 5.0 and m/z tolerance of 10 ppm. Chromatogram deconvolution was done using ADAP algorithm and chromatograms were deisotoped. Features with <2 isotopes were removed using the peaks row filter option. Variations in feature retention time between replicates were corrected using the Join Aligner algorithm (m/z tolerance, 0.01 Da; RT tolerance, 1 min). To compensate for missing peaks, the GAP filling algorithm was used at the same RT and m/z range with an m/z tolerance of 0.01 Da. Peaks present in samples defined as initial time were removed from samples defined as final time using the peaks row filter option and manual inspection after GAP filling. Features detected in at least 2 replicates in final time samples were kept for further processing. A 10-fold difference in the area under the curve mean (triplicate analysis) of each feature was selected as a cut-off between initial and final time samples. As an additional step to filter out contaminants, an in-house library list of contaminant peaks was used. Finally, peaks with MS/MS data were exported (.MGF file for GNPS) for further analysis.

MGF files were analyzed by the Global Natural Products Social Molecular Networking (GNPS) Data Analysis workflow (<https://gnps.ucsd.edu)> to create a molecular network (4). The data were filtered by removing all MS/MS peaks within +/- 17 Da of the precursor m/z. MS/MS spectra were window filtered by choosing only the top six peaks in the +/- 50Da window throughout the spectrum. The data was then clustered with MS-Cluster with a parent mass tolerance of 0.02 Da and a MS/MS fragment ion tolerance of 0.02 Da to create consensus spectra. A network was then created where edges were filtered to have a cosine score above 0.6 and more than 5 matched peaks. Further edges between two nodes were kept in the network if and only if each of the nodes appeared in each other's respective top 10 most similar nodes. The spectra in the network were then searched against GNPS' spectral libraries. The library spectra were filtered in the same manner as the input data. All matches kept between network spectra and library spectra were required to have a score above 0.7 and at least 5 matched peaks. Molecular networking data was visualized using Cytoscape 3.6.1 (5). The generated molecular network was further analyzed using the Network Annotation Propagation (NAP) tool to annotate, by *in silico* predictions, candidates for individual spectra and predict the chemical class of molecular networks or node clusters (based on the most predominant chemical class within a network) (6). The parameters used for NAP were as follows: ten first candidates for consensus score, positive acquisition mode, exact mass searches within 10 ppm and GNPS and HMDB databases.

Furthermore, the clustered .mgf file generated by GNPS was subjected to MS2LDA (<https://MS2LDA.org)> (7) for extracting Mass2motifs (M2M). MS2LDA tool allows to discover (in an unsupervised manner) groups of neutral losses and mass fragments termed M2M linked to substructures within the datasets. The parameters used were set as follows: input format .mgf, *m/z* tolerance 5.00 pm, minimum MS2 intensity 0.1 a.u., number of iterations 1000, and number of M2M 80.

A selected group of M2M were further analyzed using mzCloud Database to putatively identify substructures related to M2M-containing fragments. Neutral losses (*m/z*) predicted by MS2LDA were searched against METLIN database using the Neutral Loss Search toolbar <https://metlin.scripps.edu/landing_page.php?pgcontent=neutral_loss_search>.

**References**

1. Holman JD, Tabb DL, Mallick P. 2014. Employing ProteoWizard to Convert Raw Mass Spectrometry Data. Curr Protoc Bioinformatics 46:13 24 1-9.

2. Pluskal T, Castillo S, Villar-Briones A, Oresic M. 2010. MZmine 2: modular framework for processing, visualizing, and analyzing mass spectrometry-based molecular profile data. BMC Bioinformatics 11:395.

3. Myers OD, Sumner SJ, Li S, Barnes S, Du X. 2017. One Step Forward for Reducing False Positive and False Negative Compound Identifications from Mass Spectrometry Metabolomics Data: New Algorithms for Constructing Extracted Ion Chromatograms and Detecting Chromatographic Peaks. Anal Chem 89:8696-8703.

4. Wang M, Carver JJ, Phelan VV, Sanchez LM, Garg N, Peng Y, Nguyen DD, Watrous J, Kapono CA, Luzzatto-Knaan T, Porto C, Bouslimani A, Melnik AV, Meehan MJ, Liu WT, Crusemann M, Boudreau PD, Esquenazi E, Sandoval-Calderon M, Kersten RD, Pace LA, Quinn RA, Duncan KR, Hsu CC, Floros DJ, Gavilan RG, Kleigrewe K, Northen T, Dutton RJ, Parrot D, Carlson EE, Aigle B, Michelsen CF, Jelsbak L, Sohlenkamp C, Pevzner P, Edlund A, McLean J, Piel J, Murphy BT, Gerwick L, Liaw CC, Yang YL, Humpf HU, Maansson M, Keyzers RA, Sims AC, Johnson AR, Sidebottom AM, Sedio BE, et al. 2016. Sharing and community curation of mass spectrometry data with Global Natural Products Social Molecular Networking. Nat Biotechnol 34:828-837.

5. Shannon P, Markiel A, Ozier O, Baliga NS, Wang JT, Ramage D, Amin N, Schwikowski B, Ideker T. 2003. Cytoscape: a software environment for integrated models of biomolecular interaction networks. Genome Res 13:2498-504.

6. da Silva RR, Wang M, Nothias LF, van der Hooft JJJ, Caraballo-Rodriguez AM, Fox E, Balunas MJ, Klassen JL, Lopes NP, Dorrestein PC. 2018. Propagating annotations of molecular networks using in silico fragmentation. PLoS Comput Biol 14:e1006089.

7. van der Hooft JJ, Wandy J, Barrett MP, Burgess KE, Rogers S. 2016. Topic modeling for untargeted substructure exploration in metabolomics. Proc Natl Acad Sci U S A 113:13738-13743.
